# The Oxygen Dilemma: A Severe Challenge for the Application of Monooxygenases?

**DOI:** 10.1002/cbic.201600176

**Published:** 2016-06-30

**Authors:** Dirk Holtmann, Frank Hollmann

**Affiliations:** ^1^ DECHEMA Research InstituteTheodor-Heuss-Allee 2560486Frankfurt am MainGermany; ^2^ Department of BiotechnologyDelft University of TechnologyJulianalaan 1362628BLDelftThe Netherlands

**Keywords:** biocatalysis, monooxygenases, oxidoreductases, oxyfunctionalization, uncoupling

## Abstract

Monooxygenases are promising catalysts because they in principle enable the organic chemist to perform highly selective oxyfunctionalisation reactions that are otherwise difficult to achieve. For this, monooxygenases require reducing equivalents, to allow reductive activation of molecular oxygen at the enzymes' active sites. However, these reducing equivalents are often delivered to O_2_ either directly or via a reduced intermediate (uncoupling), yielding hazardous reactive oxygen species and wasting valuable reducing equivalents. The oxygen dilemma arises from monooxygenases' dependency on O_2_ and the undesired uncoupling reaction. With this contribution we hope to generate a general awareness of the oxygen dilemma and to discuss its nature and some promising solutions.

## 1 The Promise of Biocatalytic Oxyfunctionalisation for Organic Synthesis

**Scheme 1:**
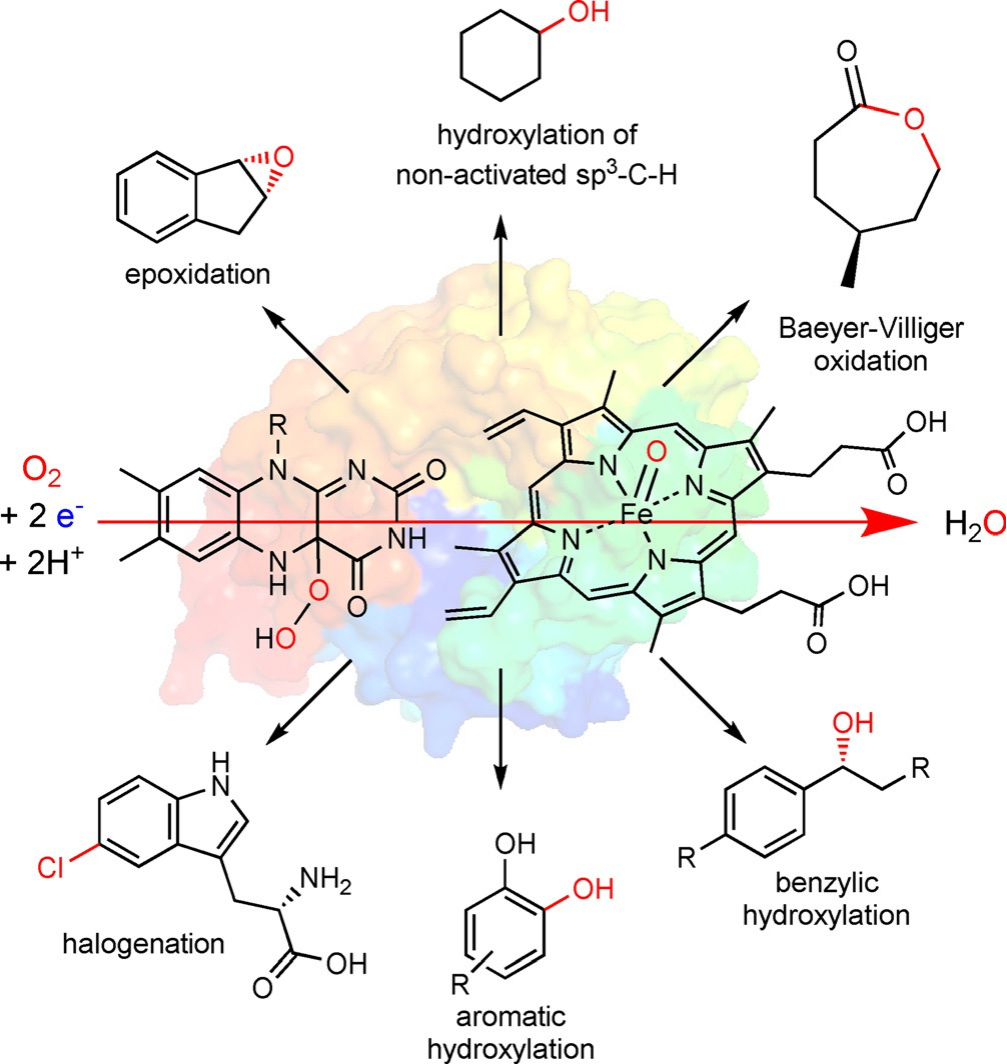
The synthetic scope of monooxygenases. Within the enzymes' active sites, highly reactive oxygen‐transfer species are generated through reductive activation of molecular oxygen. The protein scaffold controls the interaction between these reactive oxygen transfer species [e.g., Fe**⋅**oxo complexes or (hydro)peroxyflavins] and the substrates, leading to highly selective oxyfunctionalisation reactions.1c

Oxidoreductases appear set to become practical catalysts for organic synthesis.^1^ Following in the path of the well‐established hydrolases,^2^ the number of industrial1a, 3 and pre‐industrial examples^4^ of biocatalytic redox reactions is increasing rapidly.1c, 5 From a synthetic point of view monooxygenases are of particular interest for the organic chemist because they succeed in balancing high reactivity (needed to activate inert C−H bonds) and selectivity (by confining the reactive oxygenating species in a well‐defined protein scaffold). Hence, monooxygenases in principle give access to selective hydroxylations, epoxidations, Baeyer–Villiger oxidations and even halogenations (Scheme 1). In comparison with their chemical counterparts, monooxygenases often excel in terms of selectivity and catalyst performance [turnover numbers (TNs) and turnover frequencies (TOFs)].^6^

Monooxygenases might develop to become practical catalysts, enabling the organic chemist to perform highly selective oxyfunctionalisation reactions that might otherwise be difficult or require extensive protection group chemistry or longer synthesis routes. There is, however, still a range of issues to be solved to make monooxygenases truly practical catalysts. Amongst these there is the oxygen dilemma, which we briefly outline in this contribution.

## 2 The Oxygen Dilemma

A monooxygenase catalyses the selective introduction of an activated, electrophilic oxygen species (most frequently a hydroperoxo species or a highly oxidised transition‐metal**⋅**oxo complex) into its substrate(s). The activated species is obtained by reductive activation of molecular oxygen at the enzyme's active site.

One consequence of this catalytic strategy is that monooxygenases need to be supplied with stoichiometric amounts of reducing equivalents for catalysis. Generally, these reducing equivalents are derived from nicotinamide cofactors. These reducing equivalents can “get lost” in different side reactions. In other words, the reducing power is diverted from the target reaction to unproductive (futile) reduction reactions. In particular, O_2_ itself is the predominant sink for reducing equivalents and the most important reason for futile side reactions. This phenomenon is well known in the scientific literature as *uncoupling* (vide infra). Traditionally, the major issue of uncoupling is believed to be the formation of reactive oxygen species (ROSs), impairing enzyme stability and cell viability. Therefore, the majority of in vitro reaction schemes also employ (enzymatic) ROS‐scavenging systems such as superoxide dismutases and catalases, which remove the different ROSs. This, however, alleviates only one aspect of uncoupling.

Another aspect of uncoupling, broached far less frequently, is the futile consumption of the co‐substrate (stoichiometric reductant), demanding unnecessary molar surpluses. Once monooxygenase‐catalysed reactions reach preparative‐scale applications, this need for additional enzymes (to destroy the ROSs), as well as for surplus reductants, can significantly impair the attractiveness of these reactions. The dilemma arises from the fact that, although it efficiently fosters the undesired uncoupling, O_2_ also cannot be omitted from the reaction schemes.

## 3 Mechanisms of Uncoupling

Uncoupling occurs mainly 1) during oxygenation at monooxygenases' active sites, and/or 2) in the delivery of reducing equivalents to these active sites through electron‐transport chains. The main mechanisms are discussed briefly below. Because of their predominance as practical catalysts, the discussion is limited to flavin‐ and haem‐dependent monooxygenases.

### 3.1 Uncoupling within enzymes' active sites

**Scheme 2:**
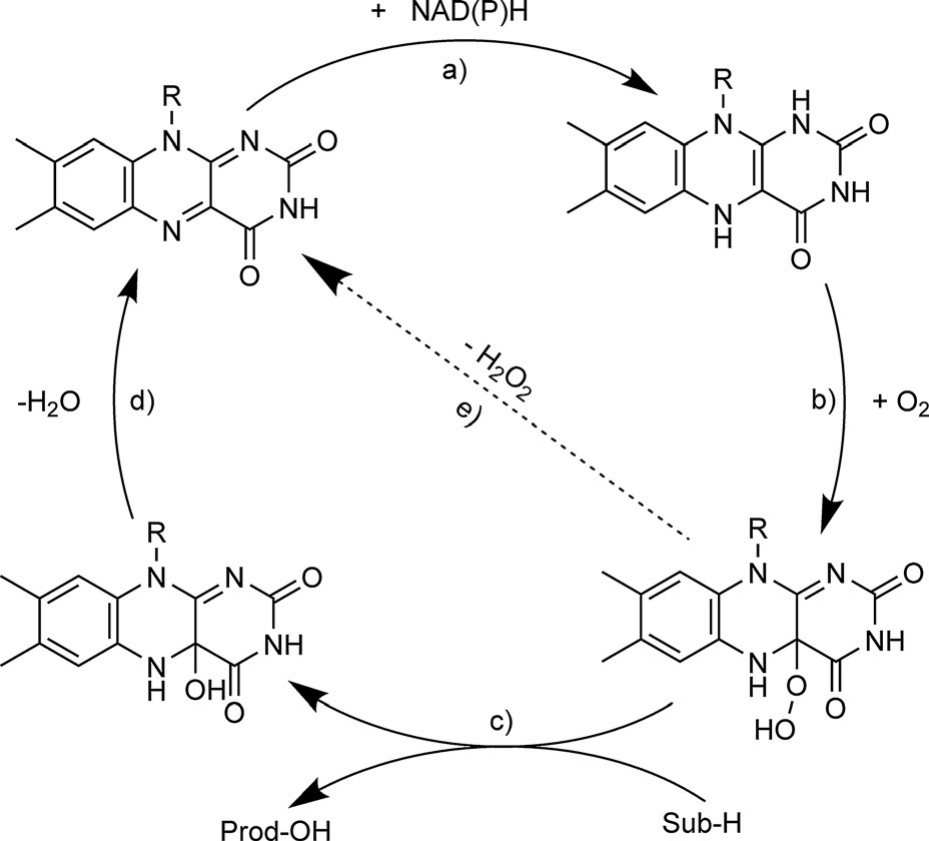
Simplified mechanism of flavin‐dependent monooxygenases, consisting of a) NAD(P)H‐dependent reduction of the flavin prosthetic group, followed by b) activation of molecular oxygen as a (hydro)peroxyflavin, and c) substrate oxygenation. The catalytic cycle is closed after d) elimination of water and reformation of the oxidised flavin. Alternatively, e) the (hydro)peroxyflavin can eliminate H_2_O_2_ spontaneously (uncoupling reaction).

*Uncoupling in the active sites of flavin‐dependent monooxygenases*: Flavin monooxygenases make use of a 4a‐(hydro)peroxo flavin (obtained after reduction of the enzyme‐bound flavin and subsequent reaction with molecular oxygen) to oxygenate their substrates. This mostly results in a 4a‐hydroxyflavin, which, after water elimination, enters into a new catalytic cycle (Scheme 2). The intermediate peroxyflavin species is believed to be stabilised by various interactions, such as by hydrogen bonding to the enzyme‐bound oxidised nicotinamide cofactor.^7^ In some cases, however, the intermediate 4a‐hydroperoxyflavin can also eliminate H_2_O_2_ directly, returning to the resting state without substrate turnover (step e in Scheme 2).^8^

The extent of uncoupling varies both with the type of flavo‐monooxygenases and with the reactions catalysed by them, as well as with the reaction conditions.^9^ Flavoprotein hydroxylases, for example, suffer significantly from uncoupling due to poor stability of the hydroperoxyflavin. Baeyer–Villiger monooxygenases, on the other hand, suffer much less from uncoupling (stable peroxyflavin).^10^

Under initial‐rate conditions (low level of conversion of the starting material), *“*uncoupling generally contributes 5–10 % of the overall NAD(P)H oxidation rate. Often the product functions as a facilitator for the uncoupling by reversibly binding to the active site and preventing productive substrate oxygenation (step c in Scheme 2). Hence, the uncoupling continuously increases with the reaction progress.^11^

Reversal of the uncoupling reaction (step e in Scheme 2) as observed with P450 monooxygenases (vide infra) has not yet been observed with flavo‐monooxygenases containing the natural cofactors. This is due to the poor electrophilicity of the oxidised flavins, resulting in low rates and unfavourable equilibria for reactions between oxidised flavins and H_2_O_2_. However, N‐alkylated flavins exhibiting higher reactivity have been successfully been integrated into flavin‐binding proteins, thereby representing a first step towards H_2_O_2_‐driven flavo‐monooxygenases.^12^

**Scheme 3:**
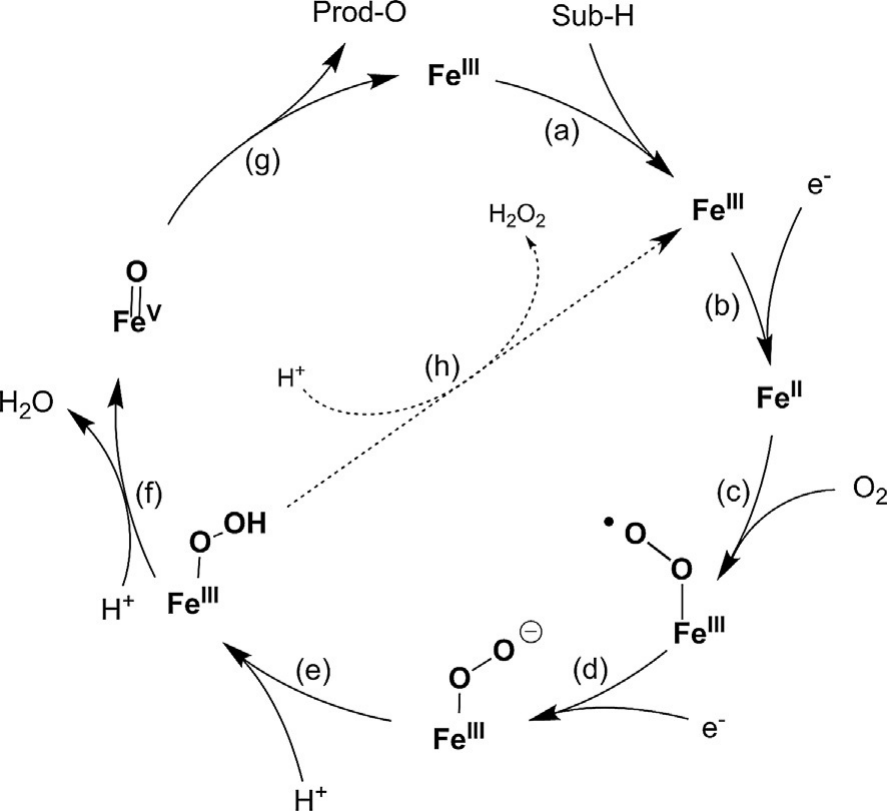
Simplified mechanism of haem‐dependent monooxygenases, consisting of two single‐electron transfer steps to haem iron (steps b and d) and formation of “compound I” (step f) to perform the oxygenation reaction (step g). The elimination of H_2_O_2_ from the intermediate Fe^III^ peroxo species is shown in step h.

*Uncoupling in the active sites of haem‐dependent monooxygenases*: Haem‐dependent monooxygenases such as the P450 monooxygenases (and non‐haem iron monooxygenases) follow a somewhat more complex mechanism than flavo‐monooxygenases, (Scheme 3).^13^

In most P450‐monooxgenase‐catalysed reactions “compound I” (step f in Scheme 3, formally an Fe^V^ oxo species but more likely an Fe^IV^ **⋅**oxo complex with a delocalised radical cation within the coordinating porphyrin ligand) represents the oxyfunctionalising species. It is formed through dehydration of an Fe**⋅**peroxo complex (step f in Scheme 3), which itself has been generated by a sequence of single‐electron transfer steps and oxygen binding (steps a–e in Scheme 3). Analogously to the previously mentioned flavin‐dependent monooxygenases, the Fe**⋅**hydroperoxo complex is also known to return to the resting state through H_2_O_2_ elimination (uncoupling, step h in Scheme 3). This step is to some extent reversible and can be exploited productively through the so‐called peroxide shunt pathway.^14^ The catalytically active compound I can also be generated directly from the resting state of a P450 monooxygenase with an organic peroxide or H_2_O_2_ (reversal of step h in Scheme 3). In principle, this represents a much simpler regeneration of P450 monooxygenases (vide infra) but it is very limited by the poor stability of the haem moiety in the presence of even low concentrations of H_2_O_2_.^15^

### 3.2 Uncoupling in the electron‐transport chain

**Scheme 4:**
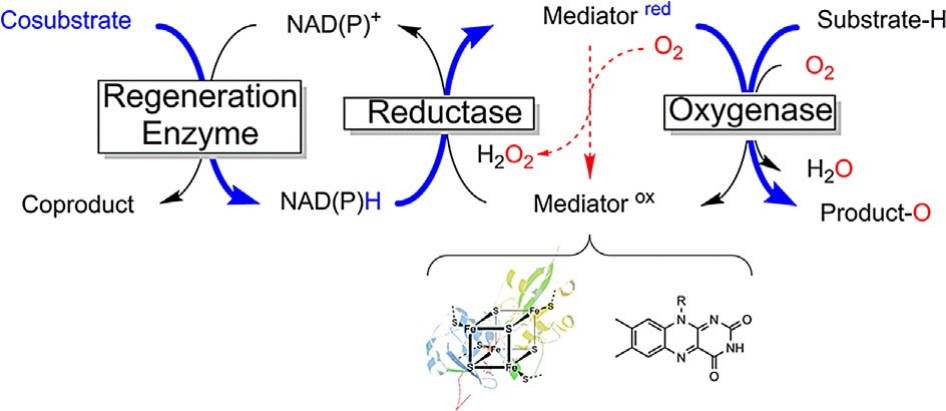
Simplified molecular architecture of multicomponent monooxygenases that are not directly dependent on NAD(P)H. Blue: path of reducing equivalents. NAD(P)H serves as a general reductant, transferring its reducing equivalents to a mediator molecule (either a flavin or an iron–sulfur cluster protein) with catalysis by a reductase. The usually protein‐based mediator delivers the reducing equivalents to the monooxygenase subunit for productive oxygen activation. However, direct reaction of the reduced mediator with O_2_ leads to futile reoxidation and (eventually) H_2_O_2_ formation.

In the previous section, uncoupling within monooxygenases' active sites has been discussed. Indeed, with many monooxygenases this also represents the major pathway of uncoupling. This is particularly true for those monooxygenases that utilise NAD(P)H directly as reductant for the prosthetic group. There are, however, also a large number of monooxygenases that obtain their reducing equivalents indirectly: that is, through a more or less complicated electron‐transport chain (Scheme 4). This section discusses uncoupling occurring within these electron‐transport chains.

*Why are some mediators inert against O_2_ whereas others are not? The nature of the oxygen dilemma*: As indicated in Scheme 4, the mediators used to shuttle reducing equivalents from NAD(P)H to monooxygenases' active sites are O_2_‐sensitive whereas NAD(P)H itself is relatively stable towards O_2_. Obviously, this raises the question of the molecular reason for this discrepancy.

**Scheme 5:**
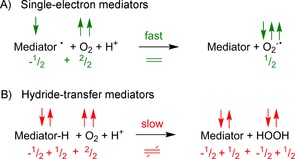
Molecular oxygen (two spins) can react quickly with single‐electron mediators because the sum of spins does not change during the reaction (spin‐allowed reaction). Hydride donors (or two‐electron mediators in general) react with molecular oxygen far more slowly because the sum of spins changes during the reaction (spin‐forbidden reaction).

A possible explanation may be found in the spin conservation rule (Wigner's rule).^16^ According to this rule, reactions during the course of which the sum of the electron spins changes are “spin‐forbidden” and therefore slow. Spin‐neutral reactions are “spin‐allowed” and fast. The electronic ground state of atmospheric oxygen is the so‐called triplet state (^3^O_2_), involving two unpaired electrons and a total spin of one (2×1/2 ). Hence, electron transfer reactions between ^3^O_2_ and single‐electron mediators are spin‐allowed because the sum of spins before and after the reaction does not change (Scheme 5, top). Reactions between ^3^O_2_ and hydride donors, on the other hand, are spin‐forbidden because the sum of spins before and after the reaction changes (Scheme 5, bottom).^17^

**Figure 1:**
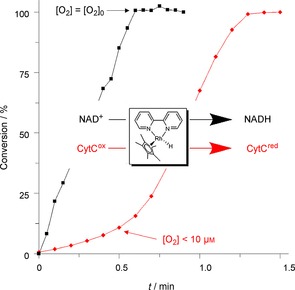
Comparison of the reduction kinetics of NAD^+^ (black) and CytC (red) with [Cp*Rh(bpy)H]^+^ in the presence of O_2_. Comparative experiments with a Clark electrode demonstrated that the dissolved O_2_ remained constant in the case of NAD^+^ reduction whereas it decreased to less than 10 μm in the first 30 s of the CytC reduction experiment.

This behaviour can nicely be exemplified with the organometallic artificial mediator [Cp*Rh(bpy)(H_2_O)]^2+^ (and its reduced form [Cp*Rh(bpy)H]^+^). Depending on the reaction conditions, this acts either as a single‐electron mediator or as a hydride‐transfer mediator (Figure 1).^18^ Whereas the reduction of NAD^+^ (hydride transfer reaction) was not influenced by the presence or absence of O_2_ the reduction of cytochrome C (single‐electron transfer) proceeded at significant rates only after the dissolved molecular oxygen had been depleted.

Hence, NAD(P)H (acting as a hydride‐transfer mediator) is metastable in the presence of atmospheric oxygen whereas reduced ferredoxins (acting as SET mediators) and reduced flavins react readily. This constitutes the oxygen dilemma.

Here it is worth briefly discussing the mechanism of flavin oxidation. Reduced flavins (FADH_2_, FMNH_2_, RfH_2_) are generally known to be very reactive with molecular oxygen. Hence, significant uncoupling can be expected in cases of those monooxygenases that depend on freely diffusing reduced flavins as mediators.

Frequently, a reoxidation mechanism in the style of the enzymatic uncoupling mechanism (Scheme 2) is proposed. However, as early as the 1970s, Massey and co‐workers intensively investigated the redox chemistry of flavins and demonstrated that, in solution, a radical‐based reoxidation mechanism most probably prevails [Eqs. ((1)) and ((2)) in comparison with Equations ((3)) and ((4))].^19^ On comparing the rate constants of both pathways [i.e., the “peroxyflavin pathway” of Equations (1) and (2)^20^ and the radical pathway of Equations (3) and (4)], it becomes clear that the latter dominates.

(1) $$\mathrm{FIH}{}^{-}+O{}_{2}+H{}^{+}\overset{k{}_{1}}{\underset{}{\to }}\mathrm{FIHOOH}$$

*k*_1_∼250 m ^−1^ s^−^

(2) $$\mathrm{FIHOOH}\overset{k{}_{2}}{\underset{}{\to }}\mathrm{FI}+H{}_{2}O{}_{2}$$

*k*_2_∼260 s^−^

(3) $$\mathrm{FIH}{}^{-}+\mathrm{FI}+H{}^{+}\overset{k{}_{3}}{\underset{k{}_{-3}}{↔}}2\;\mathrm{FIH}{}^{•}$$

*k*_3_∼1×10^6^ m ^−1^ s^−^; *k*_−3_∼5×10^8^ m ^−1^ s^−^

(4) $$\mathrm{FIH}{}^{•}+O{}_{2}\overset{k{}_{4}}{\underset{}{\to }}\mathrm{FI}+O{}_{2}{}^{•}{}^{-}+H{}^{+}$$

*k*_4_∼∼1×10^4^–1×10^6^ m ^−1^ s^−^ (depending on pH)

(5) $$\mathrm{FIH}{}^{•}+O{}_{2}{}^{•}{}^{-}+H{}^{+}\overset{k{}_{5}}{\underset{}{\to }}\mathrm{FI}+H{}_{2}O{}_{2}$$

*k*_5_∼1×10^6^ m ^−1^ s^−^

(6) $$2\;O{}_{2}{}^{•}{}^{-}+2\;H{}^{+}\overset{k{}_{6}}{\underset{}{\to }}H{}_{2}O{}_{2}+O{}_{2}$$

*k*_6_∼8×10^7^ m ^−1^ s^−^

*Flavin‐dependent monooxygenases* can be classified according to the electron donor providing the reducing equivalents needed for the reductive activation of molecular oxygen (Table 1).8b, 9a, 9c

**Table 1:** Classification of flavo‐monooxygenases according to their electron donors and prosthetic groups.

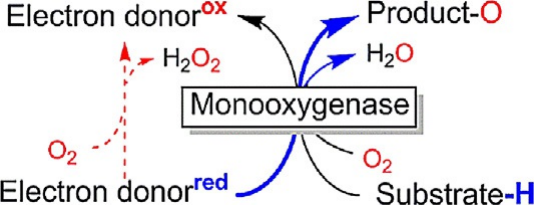			
Group	Prosthetic group	Electron donor	Typical reaction
A	FAD	NAD(P)H	hydroxylation/sufoxidation
B	FAD	NAD(P)H	Baeyer–Villiger oxidation/heteroatom oxyfunctionalisation
C	FMN	FMNH_2_	light emission and various oxyfunctionalisations
D	FAD/FMN	FADH_2_/FMNH_2_	hydroxylation
E	FAD	FADH_2_	epoxidation
F	FAD	FADH_2_	halogenation
G	FAD	substrate	oxidative decarboxylation
H	FMN	substrate	oxidative decarboxylation

Modified from van Berkel and co‐workers.8b, 9a, 9c

Groups A and B consist of enzymes that rely on NAD(P)H as external electron donor. Groups C–F are two‐protein systems, composed in each case of a monooxygenase and a flavin reductase. Groups G and H contain internal monooxygenases that reduce the flavin cofactor through substrate oxidation. With respect to the oxygen dilemma, groups A, B, G and H, on the one hand, and groups C–F, on the other, should be distinguished. The first ones (A, B, G, H) rely on O_2_‐stable reductants whereas the reductants of the latter series (C–F) are highly reactive with O_2_. Hence, especially for the latter set of enzymes, uncoupling not only occurs within the active sites (as discussed above) but also in the electron‐transport chain.

The molecular reason for the (seemingly unnecessarily) more complicated electron‐transport chains in the cases of some flavin‐dependent monooxygenases^21^ remains mysterious, especially when the additional loss of valuable reducing equivalents is considered.

**Scheme 6:**
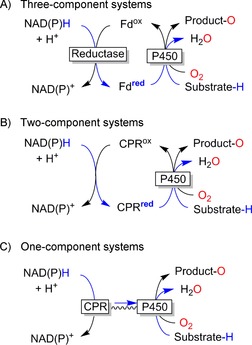
Classification of P450 monooxygenases. Fd: Ferredoxin. CPR: cytochrome P450 reductase.

*P450 monooxygenases*: In the case of haem‐dependent monooxygenases the complicated electron‐transport chain is a mechanistic requirement: NAD(P)H serves exclusively as hydride donor whereas the mechanism of haem monooxygenases involves two individual SET steps. Therefore, a relay system transforming a hydride‐transfer step into two sequential single‐electron‐transfer steps is necessary to link P450 monooxygenases to the microbial [NAD(P)H] energy metabolism. P450 monooxygenases can be classified according to the molecular architecture of the electron‐transport chain into one‐, two‐ and three‐component P450 monooxygenases (Scheme 6).^22^ They all have in common that a flavin‐dependent (FAD or FMN) reductase catalyses the initial oxidation of NAD(P)H and enables two successive SET steps. In the cases of the one‐ and two‐component P450 monooxygenases these SETs occur directly to the monooxygenase subunit, whereas in that of the three‐component systems the reductase reduces a freely diffusing ferredoxin. The reduced ferredoxin then delivers two electrons in two SETs to the monooxygenase subunit. A prototype of bacterial P450 systems is the P450cam system from *Pseudomonas putida*, in which the cytochrome catalyses the hydroxylation reaction. Furthermore, the system incorporates a FAD‐containing reductase as well as an iron–sulfur protein (putidaredoxin). This [2 Fe–2 S] ferredoxin plays the role of an electron shuttle, transferring the two electrons one at a time from putidaredoxin reductase to P450cam. Therefore, the putidaredoxin can be regarded as a natural mediator in this three‐component system.

Therefore, it can be expected that the two‐ and three‐component P450 monooxygenases (in which electron supply relies on diffusional electron mediators) in particular should be especially prone to the oxygen dilemma. The resulting uncoupling is well known in the literature and has been discussed in several review articles.^23^ Fasan recently pointed out that for many P450 monooxygenases the coupling efficiency with the natural substrate(s) can be as high as 90–98 %23b but can decrease significantly to almost any value ranging from <1 % to 30–40 % in the presence of unnatural substrates. It might be possible to overcome this issue by improving the interaction between the haem moiety and the mediator. The electron transfer between the electron‐transport proteins and the monooxygenase subunit is essential and has to be fine‐tuned to allow for efficient (that is, productive) oxygen activation and product formation.^24^

The P450 monooxygenase from *Bacillus megaterium* (P450 BM3) constituted the first example of a self‐sufficient P450 monooxygenase, gathering all electron‐transport components in one single polypeptide.^25^ The sometimes very high coupling efficiencies observed in P450 BM3 have inspired researchers to construct artificial fusion proteins. Some successful examples of this strategy have been summarised by Hlavica.^26^ However, it should also be mentioned here that this approach does not always lead to improved self‐sufficient enzymes. Even in P450 BM3 the uncoupling can be as high as 60 % or even more.^27^

Overall, it can be concluded that uncoupling in monooxygenases occurs naturally. In the case of haem‐dependent monooxygenases in which single‐electron transfers are a mechanistic necessity, uncoupling is even inevitable. This raises the question of why natural oxygenation schemes would rely on such wasteful and dangerous processes. One possible explanation might be that the uncoupling generated by monooxygenases (and their electron‐transport chains) carries no weight in comparison with mitochondrial uncoupling^28^ or the uncoupling occurring in chloroplasts.^29^ Hence, the oxygen dilemma might be just the price to pay for an aerobic life.

## 4 Ways to Deal with the Oxygen Dilemma

Essentially, the oxygen dilemma is a kinetic phenomenon. Reducing equivalents can either be delivered to the monooxygenases productively or they can be diverted to free molecular oxygen, thereby wasting valuable reducing equivalents and yielding ROSs. Approaches to alleviation of the oxygen dilemma should therefore focus on increasing the rate of the first (desired) pathway while slowing down the latter (undesired) reaction.

To assuage uncoupling within the monooxygenases' active sites, protein engineering appears to be the method of choice. Especially for the flavo‐monooxygenases, structural information about the stabilisation of the reactive 4a‐(hydro)peroxyflavin is available7a, 7b, 30 and can serve as starting point to improve the efficiency of other flavo‐monooxygenases. The negative effect of accumulating product on the uncoupling can best be addressed by in situ product removal strategies, maintaining the product concentration in the aqueous reaction mixture low and facilitating downstream processing.^11^, ^31^

Improving the coupling efficiency of the electron‐transport chains in multiple‐component monooxygenases can be achieved by improving the interaction between the reduced natural mediators and the monooxygenase subunit. In the case of P450 monooxygenases the concept of molecular building blocks—that is, combining monooxygenases with the most suitable mediators and reductases for efficient interaction and electron transfer—might represent a future approach.^24^ Similar trials with two‐component flavo‐monooxygenase have been reported.^32^

**Scheme 7:**
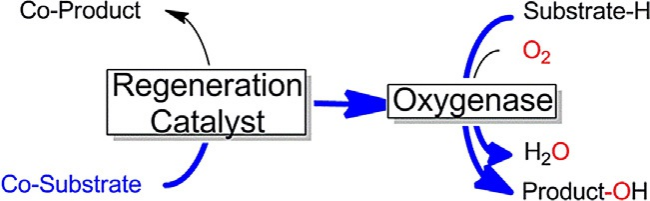
Simplified regeneration of (mono)oxygenases by direct reductive regeneration of the enzymes' active sites.

Another line of research involves simplified electron‐transport chains. Ideally, one single catalyst functions as relay system between a sacrificial electron donor and a monooxygenase (Scheme 7), thereby substituting the nicotinamide cofactor together with the corresponding reductases and mediators. The promise of this approach lies in its high degree of simplification and, hopefully, greater robustness of this reaction scheme.

The most common regeneration catalysts are transition‐metal complexes such as cobalt sepulchrate,^33^ cobaltocene,^34^ or ruthenium^35^ or rhodium complexes.21d, 36 Organic mediators such as flavins^37^ or redox‐active dyes^38^ have also been reported. Unfortunately, little information about the coupling efficiency can be found in most of these contributions. However, from the few cases in which the uncoupling is quantified it becomes clear that classical one‐electron mediators are rather poor in their coupling efficiency whereas hydride donors are usually significantly better.

An additional complication occurs in the case of electrochemical reaction systems.36b, 37e, 39 Generally, the cathode potentials required for efficient reduction of the mediator (natural or artificial) used are more negative than the O_2_ reduction potential. Hence, direct cathodic O_2_ reduction occurs during the electrolyses, reducing the current yield (significantly contributing to uncoupling) and generating ROSs.

Several approaches to dealing with the oxygen dilemma in artificial regeneration systems have been proposed. Of these, a few (promising) strategies are discussed below.

**Scheme 8:**
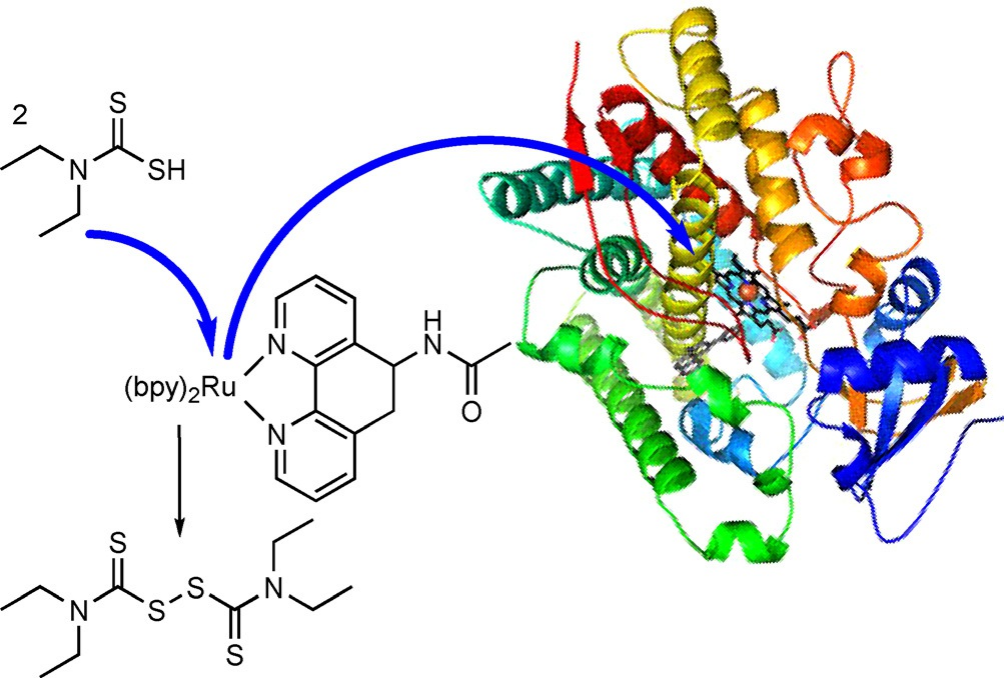
Covalent linkage of a Ru‐photosensitzer/mediator to a P450 monooxygenase to facilitate electron transfer.

Cheruzel and co‐workers have reported covalent linkages between P450 monooxygenases and (mostly Ru‐based) photosensitisers/mediators with great improvement in the reaction rates. Possibly, this can also be attributed—at least to some extent—to decreased futile reoxidation of the reduced mediators due to elimination of diffusion limitations (Scheme 8).35a, 35c, 40

The previously mentioned direct cathodic reduction of O_2_ can in principle be alleviated by choice of a suitable reactor design minimising the O_2_ concentration in the cathode compartment.37d, 39a, 39b, 41 Similarly, engineering the monooxygenase mediator interaction on the basis of rational design might improve the efficiency of electron transfer.33e, 42 Another possible handle could be the choice of electrode material. A recent study, for example, demonstrated that glassy carbon exhibited significantly higher reduction rates with Co sepulchrate than Pt electrodes, together with reduced O_2_ reduction rates.^43^

**Scheme 9:**
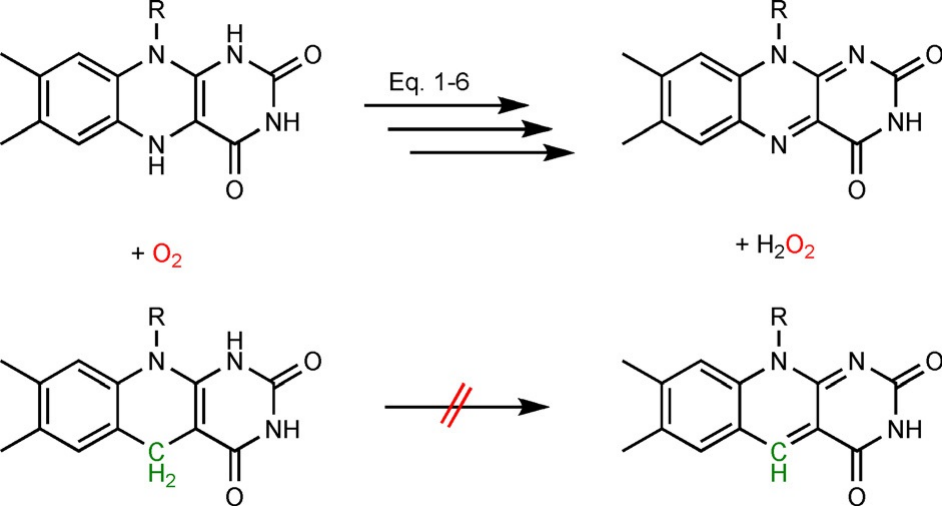
5‐Deazaflavins (bottom) as O_2_‐stable reduced mediators (in comparison with normal flavins, top).

Employing O_2_‐stable artificial mediators could be a viable approach. For example, deazaflavins, which have been known for decades as O_2_‐resistant analogues of the common flavin mediators,^19^, ^44^ have been used as photocatalysts/mediators to regenerate P450 BM3 and Old Yellow Enzymes. In both cases O_2_ efficiently interfered in the reduction of the enzyme prosthetic group (haem or FMN). Changing the mediator from a normal flavin to a deazaflavin analogue increased the efficiency of the reaction schemes significantly (Scheme 9).37b, 45

**Scheme 10:**
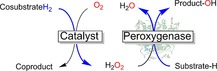
Simplified scheme for peroxygenase‐catalysed oxyfunctionalisation reactions exploiting the oxygen dilemma.

Finally, it is also worth mentioning that “exploiting” the oxygen dilemma by using H_2_O_2_‐dependent enzymes is a viable and very promising solution (Scheme 10).

Peroxygenases utilise H_2_O_2_ to generate the catalytically active compound I, known from P450 chemistry (reversal of step h in Scheme 3), directly.^46^ Fortunately, the number of synthetically interesting peroxygenases is increasing rapidly,^47^ and new protein engineering tools to adjust the substrate scope and selectivity of these enzymes are available.^48^ The issue of poor robustness of these haem‐dependent enzymes against H_2_O_2_ can be addressed by various catalytic in situ H_2_O_2_ generation systems.^49^ Therefore, exciting new biocatalytic oxyfunctionalisation reactions can be expected in the near future.

## 5 Conclusions

The oxygen dilemma is a reality that has to be faced in biocatalytic oxyfunctionalisation chemistry. The high reactivity of molecular (triplet) oxygen with the most common natural and man‐made redox mediators interferes in most electron‐transport chains delivering electrons to monooxygenases. Today, the major challenge of this well‐known uncoupling is seen in the formation of reactive oxygen species (ROSs) and their negative influence on the robustness of the (bio)catalysts used. This issue is relatively easily alleviated by using nature's arsenal of ROS‐scavenging enzymes (such as superoxide dismutases and catalases).

In view of the fact that monooxygenases today are mostly used in the fine chemicals and pharmaceutical sectors, the wasteful nature of uncoupling is of lesser importance.^50^ If large‐scale applications of monooxygenases are envisioned, however, the waste of valuable reduction equivalents comes to the fore. From the economic point of view, bulk products with lower margins do not allow for wasteful processes. Also from an environmental point of view, though, futile waste of resources has to be avoided.^51^

A first step towards more redox‐efficient processes should be a quantitative understanding of the uncoupling. More experimental data quantifying the coupling efficiency of monooxygenase reactions (not only from initial rate experiments but also throughout the processes) should be helpful in creating a general awareness of the oxygen dilemma. In this contribution a few promising approaches have been mentioned. We hope that this minireview might encourage other researchers to focus on solving the oxygen dilemma to make biocatalytic oxyfunctionalisation truly efficient and practical.

## References

[1a] U. T. Bornscheuer , G. W. Huisman , R. J. Kazlauskas , S. Lutz , J. C. Moore , K. Robins , Nature 2012, 485, 185–194; 22575958 10.1038/nature11117

[1b] F. Hollmann , I. W. C. E. Arends , D. Holtmann , Green Chem. 2011, 13, 2285–2313;

[1c] F. Hollmann , I. W. C. E. Arends , K. Buehler , A. Schallmey , B. Buhler , Green Chem. 2011, 13, 226–265.

[2a] A. Liese , K. Seelbach , C. Wandrey , Industrial Biotransformations, Wiley-VCH, Weinheim, 2006;

[2b] U. Bornscheuer , R. Kazlauskas , Hydrolases in Organic Synthesis, 2nd ed., Wiley-VCH, Weinheim, 2006.

[3] G. W. Huisman , J. Liang , A. Krebber , Curr. Opin. Chem. Biol. 2010, 14, 122–129. 20071211 10.1016/j.cbpa.2009.12.003

[4] D. Holtmann , M. W. Fraaije , D. J. Opperman , I. W. C. E. Arends , F. Hollmann , Chem. Commun. 2014, 50, 13180–13200. 10.1039/c3cc49747j 24902635

[5] K. Drauz, H. Groeger, O. May, *Enzyme Catalysis in Organic Synthesis*, Wiley-VCH, Weinheim, **2012**.

[6] E. Roduner , W. Kaim , B. Sarkar , V. B. Urlacher , J. Pleiss , R. Gläser , W.-D. Einicke , G. A. Sprenger , U. Beifuß , E. Klemm , C. Liebner , H. Hieronymus , S.-F. Hsu , B. Plietker , S. Laschat , ChemCatChem 2013, 5, 82–112.

[7a] E. Malito , A. Alfieri , M. W. Fraaije , A. Mattevi , Proc. Natl. Acad. Sci. USA 2004, 101, 13157–13162; 15328411 10.1073/pnas.0404538101 PMC516541

[7b] A. Alfieri , E. Malito , R. Orru , M. W. Fraaije , A. Mattevi , Proc. Natl. Acad. Sci. USA 2008, 105, 6572–6577; 18443301 10.1073/pnas.0800859105 PMC2373336

[7c] R. Orru , H. M. Dudek , C. Martinoli , D. E. Torres Pazmino , A. Royant , M. Weik , M. W. Fraaije , A. Mattevi , J. Biol. Chem. 2011, 286, 29284–29291. 21697090 10.1074/jbc.M111.255075 PMC3190734

[8a] M. J. H. Moonen , M. W. Fraaije , I. M. C. M. Rietjens , C. Laane , W. J. H. van Berkel , Adv. Synth. Catal. 2002, 344, 1023–1035;

[8b] W. J. H. van Berkel , N. M. Kamerbeek , M. W. Fraaije , J. Biotechnol. 2006, 124, 670–689; 16712999 10.1016/j.jbiotec.2006.03.044

[8c] G. Gygli , W. J. H. Van Berkel , Curr. Biotechnol. 2015, 4, 100–110.

[9a] M. M. E. Huijbers , S. Montersino , A. H. Westphal , D. Tischler , W. J. H. van Berkel , Arch. Biochem. Biophys. 2014, 544, 2–17; 24361254 10.1016/j.abb.2013.12.005

[9b] B. A. Palfey , C. A. McDonald , Arch. Biochem. Biophys. 2010, 493, 26–36; 19944667 10.1016/j.abb.2009.11.028

[9c] S. Montersino , D. Tischler , G. T. Gassner , W. J. H. van Berkel , Adv. Synth. Catal. 2011, 353, 2301–2319;

[9d] B. Entsch , W. J. H. Van Berkel , FASEB J. 1995, 9, 476–483; 7737455 10.1096/fasebj.9.7.7737455

[9e] A. P. Jadan , M. J. H. Moonen , S. Boeren , L. A. Golovleva , I. Rietjens , W. J. H. van Berkel , Adv. Synth. Catal. 2004, 346, 367–375.

[10] C. A. McDonald , R. L. Fagan , F. Collard , V. M. Monnier , B. A. Palfey , J. Am. Chem. Soc. 2011, 133, 16809–16811. 21958058 10.1021/ja2081873 PMC3203534

[11a] W. A. Suske , M. Held , A. Schmid , T. Fleischmann , M. G. Wubbolts , H.-P. E. Kohler , J. Biol. Chem. 1997, 272, 24257–24265; 9305879 10.1074/jbc.272.39.24257

[11b] M. Held , W. Suske , A. Schmid , K. H. Engesser , H. P. E. Kohler , B. Witholt , M. G. Wubbolts , J. Mol. Catal. B 1998, 5, 87–93;

[11c] M. Held , A. Schmid , H. P. E. Kohler , W. Suske , B. Witholt , M. G. Wubbolts , Biotechnol. Bioeng. 1999, 62, 641–648; 9951522 10.1002/(sici)1097-0290(19990320)62:6<641::aid-bit3>3.0.co;2-h

[11d] A. Schmid , I. Vereyken , M. Held , B. Witholt , J. Mol. Catal. B 2001, 11, 455–462.

[12a] G. de Gonzalo , M. W. Fraaije , ChemCatChem 2012, 5, 403–415;

[12b] G. de Gonzalo , C. Smit , J. F. Jin , A. J. Minnaard , M. W. Fraaije , Chem. Commun. 2011, 47, 11050–11052. 10.1039/c1cc14039f 21901197

[13a] L. M. Blank , B. E. Ebert , K. Buehler , B. Bühler , Antioxid. Redox Signaling. 2010, 13, 349–394; 10.1089/ars.2009.2931 20059399

[13b] M. K. Julsing , S. Cornelissen , B. Buhler , A. Schmid , Curr. Opin. Chem. Biol. 2008, 12, 177–186. 18258209 10.1016/j.cbpa.2008.01.029

[14a] E. G. Hrycay , J. A. Gustafsson , M. Ingelman-Sundberg , L. Ernster , Biochem. Biophys. Res. Commun. 1975, 66, 209–216; 240357 10.1016/s0006-291x(75)80315-9

[14b] H. Joo , Z. Lin , F. H. Arnold , Nature 1999, 399, 670–673; 10385118 10.1038/21395

[14c] S. T. Jung , R. Lauchli , F. H. Arnold , Curr. Opin. Biotechnol. 2011, 22, 809–817. 21411308 10.1016/j.copbio.2011.02.008 PMC3118264

[15] B. Valderrama , M. Ayala , R. Vazquez-Duhalt , Chem. Biol. 2002, 9, 555–565. 12031662 10.1016/s1074-5521(02)00149-7

[16] A. D. McNaught, A. Wilkinson, *IUPAC Compendium of Chemical Terminology*, Wiley-Blackwell, Oxford, **2014**, DOI: 10.1351/goldbook.S05701.

[17] This is also the reason why organic matter is usually metastable under atmospheric oxygen. The thermodynamically feasible oxidation reaction is kinetically impaired due to this rule. On the other hand, this also explains the high reactivity of excited singlet oxygen ^1^O_2_ with two paired spins.

[18] F. Hollmann , A. Schmid , J. Inorg. Biochem. 2009, 103, 313–315. 19087893 10.1016/j.jinorgbio.2008.11.005

[19] V. Massey , J. Biol. Chem. 1994, 269, 22459–22462. 8077188

[20] Note that the reaction in Equation (1) is spin-forbidden, whereas all of the other reactions are spin-allowed.

[21a] K. Otto , K. Hofstetter , M. Rothlisberger , B. Witholt , A. Schmid , J. Bacteriol. 2004, 186, 5292–5302; 15292130 10.1128/JB.186.16.5292-5302.2004 PMC490909

[21b] D. Tischler , R. Kermer , J. A. D. Groning , S. R. Kaschabek , W. J. H. van Berkel , M. Schlomann , J. Bacteriol. 2010, 192, 5220–5227; 20675468 10.1128/JB.00723-10 PMC2944547

[21c] H. Toda , R. Imae , N. Itoh , Tetrahedron: Asymmetry 2012, 23, 1542–1549;

[21d] S. Unversucht , F. Hollmann , A. Schmid , K.-H. van Pée , Adv. Synth. Catal. 2005, 347, 1163–1167;

[21e] C. S. Neumann , C. T. Walsh , R. R. Kay , Proc. Natl. Acad. Sci. USA 2010, 107, 5798–5803; 20231486 10.1073/pnas.1001681107 PMC2851905

[21f] J. T. Payne , M. C. Andorfer , J. C. Lewis , Angew. Chem. Int. Ed. 2013, 52, 5271–5274; 10.1002/anie.201300762 PMC3824154 23592388

[21g] D. R. M. Smith , S. Gruschow , R. J. M. Goss , Curr. Opin. Chem. Biol. 2013, 17, 276–283. 23433955 10.1016/j.cbpa.2013.01.018

[22a] V. B. Urlacher , M. Girhard , Trends Biotechnol. 2012, 30, 26–36; 21782265 10.1016/j.tibtech.2011.06.012

[22b] F. Hannemann , A. Bichet , K. M. Ewen , R. Bernhardt , Biochim. Biophys. Acta Gen. Subj. 2007, 1770, 330–344. 10.1016/j.bbagen.2006.07.017 16978787

[23a] R. Bernhardt , V. B. Urlacher , Appl. Microbiol. Biotechnol. 2014, 98, 6185–6203; 24848420 10.1007/s00253-014-5767-7

[23b] R. Fasan , ACS Catal. 2012, 2, 647–666;

[23c] E. O'Reilly , V. Kohler , S. L. Flitsch , N. J. Turner , Chem. Commun. 2011, 47, 2490–2501; 10.1039/c0cc03165h 21264369

[23d] E. G. Funhoff , J. B. Van Beilen , Biocatal. Biotransform. 2007, 25, 186–193.

[24] G. Gilardi , Y. T. Meharenna , G. E. Tsotsou , S. J. Sadeghi , M. Fairhead , S. Giannini , Biosens. Bioelectron. 2002, 17, 133–145. 11742744 10.1016/s0956-5663(01)00286-x

[25] L. O. Narhi , A. J. Fulco , J. Biol. Chem. 1986, 261, 7160–7169. 3086309

[26] P. Hlavica , Biotechnol. Adv. 2009, 27, 103–121. 18976700 10.1016/j.biotechadv.2008.10.001

[27] J. B. Lim , K. A. Barker , K. A. Eller , L. Jiang , V. Molina , J. F. Saifee , H. D. Sikes , Protein Sci. 2015, 24, 1874–1883. 26311413 10.1002/pro.2793 PMC4622219

[28] M. D. Brand , Exp. Gerontol. 2000, 35, 811–820. 11053672 10.1016/s0531-5565(00)00135-2

[29] B. Ivanov , M. Kozuleva , M. Mubarakshina , in Cell Metabolism: Cell Homeostasis and Stress Response (Ed.: P. Bubulya), 2012, pp. 39–72.

[30a] R. Baron , C. Riley , P. Chenprakhon , K. Thotsaporn , R. T. Winter , A. Alfieri , F. Forneris , W. J. H. van Berkel , P. Chaiyen , M. W. Fraaije , A. Mattevi , J. A. McCammon , Proc. Natl. Acad. Sci. USA 2009, 106, 10603–10608; 19541622 10.1073/pnas.0903809106 PMC2698890

[30b] N. G. H. Leferink , M. W. Fraaije , H. J. Joosten , P. J. Schaap , A. Mattevi , W. J. H. van Berkel , J. Biol. Chem. 2009, 284, 4392–4397. 19088070 10.1074/jbc.M808202200

[31] I. Hilker , M. C. Gutierrez , R. Furstoss , J. Ward , R. Wohlgemuth , V. Alphand , Nat. Protoc. 2008, 3, 546–554. 18323823 10.1038/nprot.2007.532

[32a] D. Tischler , J. A. D. Groning , S. R. Kaschabek , M. Schlomann , Appl. Biochem. Biotechnol. 2012, 167, 931–944; 22528652 10.1007/s12010-012-9659-y

[32b] D. Tischler , M. Schlomann , W. J. H. van Berkel , G. T. Gassner , FEBS Lett. 2013, 587, 3848–3852. 24157359 10.1016/j.febslet.2013.10.013 PMC3858207

[33a] U. Schwaneberg , D. Appel , J. Schmitt , R. D. Schmid , J. Biotechnol. 2000, 84, 249–257; 11164266 10.1016/s0168-1656(00)00357-6

[33b] L. Zhao , G. Güven , Y. Li , U. Schwaneberg , Appl. Microbiol. Biotechnol. 2011, 91, 989–999; 21562982 10.1007/s00253-011-3290-7

[33c] S. Z. Çekiç , D. Holtmann , G. Güven , K.-M. Mangold , U. Schwaneberg , J. Schrader , Electrochem. Commun. 2010, 12, 1547–1550;

[33d] C. Ley , H. Schewe , F. W. Strohle , A. J. Ruff , U. Schwaneberg , J. Schrader , D. Holtmann , J. Mol. Catal. B 2013, 92, 71–78;

[33e] F. W. Ströhle , S. Z. Cekic , A. O. Magnusson , U. Schwaneberg , D. Roccatano , J. Schrader , D. Holtmann , J. Mol. Catal. B 2013, 88, 47–51.

[34] A. K. Udit , F. H. Arnold , H. B. Gray , J. Inorg. Biochem. 2004, 98, 1547–1550. 15337607 10.1016/j.jinorgbio.2004.06.007

[35a] M. Kato, Q. Lam, M. Bhandarkar, T. Banh, J. Heredia, A. U. L. Cheruzel, *Comp. Rend. Chim* **2016**, DOI: 10.1016/j.crci.2015.10.005;

[35b] R. Sevrioukova , C. E. Immoos , T. L. Poulos , Isr. J. Chem. 2000, 40, 47–53;

[35c] N.-H. Tran , N. Huynh , T. Bui , Y. Nguyen , P. Huynh , M. E. Cooper , L. E. Cheruzel , Chem. Commun. 2011, 47, 11936–11938. 10.1039/c1cc15124j PMC3213755 21975564

[36a] F. Hollmann , P. C. Lin , B. Witholt , A. Schmid , J. Am. Chem. Soc. 2003, 125, 8209–8217; 12837091 10.1021/ja034119u

[36b] F. Hollmann , A. Schmid , E. Steckhan , Angew. Chem. Int. Ed. 2001, 40, 169–171; 10.1002/1521-3773(20010105)40:1<169::AID-ANIE169>3.0.CO;2-T 29711966

[37a] V. V. Shumyantseva , T. V. Bulko , R. D. Schmid , A. I. Archakov , Biosens. Bioelectron. 2002, 17, 233–238; 11839477 10.1016/s0956-5663(01)00181-6

[37b] F. E. Zilly , A. Taglieber , F. Schulz , F. Hollmann , M. T. Reetz , Chem. Commun. 2009, 7152–7154; 10.1039/b913863c 19921013

[37c] M. Girhard , E. Kunigk , S. Tihovsky , V. V. Shumyantseva , V. B. Urlacher , Biotechnol. Appl. Biochem. 2013, 60, 111–118; 23586998 10.1002/bab.1063

[37d] R. Ruinatscha , C. Dusny , K. Buehler , A. Schmid , Adv. Synth. Catal. 2009, 351, 2505–2515;

[37e] F. Hollmann , K. Hofstetter , T. Habicher , B. Hauer , A. Schmid , J. Am. Chem. Soc. 2005, 127, 6540–6541; 15869268 10.1021/ja050997b

[37f] C. E. Paul , D. Tischler , A. Riedel , T. Heine , N. Itoh , F. Hollmann , ACS Catal. 2015, 5, 2961–2965.

[38] J. H. Park , S. H. Lee , G. S. Cha , D. S. Choi , D. H. Nam , J. H. Lee , J.-K. Lee , C.-H. Yun , K. J. Jeong , C. B. Park , Angew. Chem. Int. Ed. 2015, 54, 969–973; 10.1002/anie.201410059 25430544

[39a] V. Reipa , M. P. Mayhew , V. L. Vilker , Proc. Natl. Acad. Sci. USA 1997, 94, 13554–13558; 9391064 10.1073/pnas.94.25.13554 PMC28344

[39b] M. P. Mayhew , V. Reipa , M. J. Holden , V. L. Vilker , Biotechnol. Prog. 2000, 16, 610–616; 10933836 10.1021/bp000067q

[39c] J. Kazlauskaite , A. C. G. Westlake , L. L. Wong , H. A. O. Hill , Chem. Commun. 1996, 2189–2190;

[39d] Y. M. Lvov , Z. Q. Lu , J. B. Schenkman , X. L. Zu , J. F. Rusling , J. Am. Chem. Soc. 1998, 120, 4073–4080;

[39e] A. Fantuzzi , M. Fairhead , G. Gilardi , J. Am. Chem. Soc. 2004, 126, 5040–5041; 15099066 10.1021/ja049855s

[39f] B. D. Fleming , D. L. Johnson , A. M. Bond , L. L. Martin , Expert Opin. Drug Metab. Toxicol. 2006, 2, 581–589; 16859406 10.1517/17425255.2.4.581

[39g] Y. Mie , M. Suzuki , Y. Komatsu , J. Am. Chem. Soc. 2009, 131, 6646–6647; 19402636 10.1021/ja809364r

[39h] S. Y. Rhieu , D. R. Ludwig , V. S. Siu , G. T. R. Palmore , Electrochem. Commun. 2009, 11, 1857–1860.

[40a] M. E. Ener , Y.-T. Lee , J. R. Winkler , H. B. Gray , L. Cheruzel , Proc. Natl. Acad. Sci. USA 2010, 107, 18783–18786; 20947800 10.1073/pnas.1012381107 PMC2973866

[40b] N.-H. Tran , N. Huynh , G. Chavez , A. Nguyen , S. Dwaraknath , T.-A. Nguyen , M. Nguyen , L. Cheruzel , J. Inorg. Biochem. 2012, 115, 50–56; 22922311 10.1016/j.jinorgbio.2012.05.012 PMC3486690

[40c] N. H. Tran , D. Nguyen , S. Dwaraknath , S. Mahadevan , G. Chavez , A. Nguyen , T. Dao , S. Mullen , T. A. Nguyen , L. E. Cheruzel , J. Am. Chem. Soc. 2013, 135, 14484–14487. 24040992 10.1021/ja409337v PMC3938315

[41a] V. Vilker , V. Reipa , M. Mayhew , M. Holden , J. Am. Oil Chem. Soc. 1999, 76, 1283–1289;

[41b] Y. V. Grinkova , I. G. Denisov , M. A. McLean , S. G. Sligar , Biochem. Biophys. Res. Commun. 2013, 430, 1223–1227. 23266608 10.1016/j.bbrc.2012.12.072 PMC4191626

[42] R. Verma , U. Schwaneberg , D. Holtmann , D. Roccatano , J. Chem. Theory Comput. 2016, 12, 353–363. 26631637 10.1021/acs.jctc.5b00290

[43] A. Tosstorff , A. Dennig , A. J. Ruff , U. Schwaneberg , V. Sieber , K. M. Mangold , J. Schrader , D. Holtmann , J. Mol. Catal. B 2014, 108, 51–58.

[44a] V. Massey , P. Hemmerich , J. Biol. Chem. 1977, 252, 5612–5614; 407227

[44b] V. Massey , P. Hemmerich , W. R. Knappe , H. J. Duchstein , H. Fenner , Biochemistry 1978, 17, 9–17; 618539

[44c] V. Massey , M. Stankovich , P. Hemmerich , Biochemistry 1978, 17, 1–8. 618535 10.1021/bi00594a001

[45] M. Mifsud Grau , J. C. van der Toorn , L. G. Otten , P. Macheroux , A. Taglieber , F. E. Zilly , I. W. C. E. Arends , F. Hollmann , Adv. Synth. Catal. 2009, 351, 3279–3286.

[46] S. Bormann , A. Gomez Baraibar , Y. Ni , D. Holtmann , F. Hollmann , Catal. Sci. Technol. 2015, 5, 2038–2052.

[47a] M. Hofrichter , R. Ullrich , M. J. Pecyna , C. Liers , T. Lundell , Appl. Microbiol. Biotechnol. 2010, 87, 871–897; 20495915 10.1007/s00253-010-2633-0

[47b] M. Hofrichter , R. Ullrich , Curr. Opin. Chem. Biol. 2014, 19, 116–125. 24607599 10.1016/j.cbpa.2014.01.015

[48a] A. T. Martínez , F. J. Ruiz-Dueñas , A. Gutiérrez , J. C. del Río , M. Alcalde , C. Liers , R. Ullrich , M. Hofrichter , K. Scheibner , L. Kalum , J. Vind , H. Lund , Biofuels Bioprod. Biorefin. 2014, 8, 819–835;

[48b] P. Molina-Espeja , E. Garcia-Ruiz , D. Gonzalez-Perez , R. Ullrich , M. Hofrichter , M. Alcalde , Appl. Environ. Microbiol. 2014, 80, 3496–3507; 24682297 10.1128/AEM.00490-14 PMC4018863

[48c] P. Molina-Espeja , M. Canellas , F. J. Plou , M. Hofrichter , F. Lucas , V. Guallar , M. Alcalde , ChemBioChem 2016, 17, 341–349; 26677801 10.1002/cbic.201500493

[48d] P. Molina-Espeja , S. Ma , D. M. Mate , R. Ludwig , M. Alcalde , Enzyme Microb. Technol. 2015, 73–74, 29–33. 10.1016/j.enzmictec.2015.03.004 26002501

[49a] D. I. Perez , M. Mifsud Grau , I. W. C. E. Arends , F. Hollmann , Chem. Commun. 2009, 6848–6850; 10.1039/b915078a 19885500

[49b] E. Churakova , M. Kluge , R. Ullrich , I. Arends , M. Hofrichter , F. Hollmann , Angew. Chem. Int. Ed. 2011, 50, 10716–10719; 10.1002/anie.201105308 21922621

[49c] C. E. Paul , E. Churakova , E. Maurits , M. Girhard , V. B. Urlacher , F. Hollmann , Bioorg. Med. Chem. 2014, 22, 5692–5696; 24984939 10.1016/j.bmc.2014.05.074

[49d] I. Zachos , S. Gassmeyer , D. Bauer , V. Sieber , F. Hollmann , R. Kourist , Chem. Commun. 2015, 51, 1918–1921; 10.1039/c4cc07276f 25531559

[49e] Y. Ni , E. Fernández-Fueyo , A. G. Baraibar , R. Ullrich , M. Hofrichter , H. Yanase , M. Alcalde , W. J. H. van Berkel , F. Hollmann , Angew. Chem. Int. Ed. 2016, 55, 798–801; 10.1002/anie.201507881 26607550

[49f] T. Krieg , S. Huttmann , K.-M. Mangold , J. Schrader , D. Holtmann , Green Chem. 2011, 13, 2686–2689;

[49g] L. Getrey , T. Krieg , F. Hollmann , J. Schrader , D. Holtmann , Green Chem. 2014, 16, 1104–1108;

[49h] D. Holtmann , T. Krieg , L. Getrey , J. Schrader , Catal. Commun. 2014, 51, 82–85.

[50] P. Tufvesson , J. Lima-Ramos , M. Nordblad , J. M. Woodley , Org. Process Res. Dev. 2011, 15, 266–274.

[51] Y. Ni , D. Holtmann , F. Hollmann , ChemCatChem 2014, 6, 930–943.

